# Effects of NPY-2 Receptor Antagonists, Semaglutide, PYY_3-36_, and Empagliflozin on Early MASLD in Diet-Induced Obese Rats

**DOI:** 10.3390/nu16060904

**Published:** 2024-03-21

**Authors:** Simon Kloock, Niklas Haerting, Gloria Herzog, Marie Oertel, Niklas Geiger, Andreas Geier, Vasco Sequeira, Alexander Nickel, Michael Kohlhaas, Martin Fassnacht, Ulrich Dischinger

**Affiliations:** 1 Department of Internal Medicine, Division of Endocrinology and Diabetes, University Hospital Wuerzburg, University of Wuerzburg, 97080 Wuerzburg, Germany; kloock_s@ukw.de (S.K.); haerting_n@ukw.de (N.H.); oertel_m@ukw.de (M.O.); geiger_n1@ukw.de (N.G.); fassnacht_m@ukw.de (M.F.); 2 Institute of Pathology, University of Wuerzburg, 97070 Wuerzburg, Germany; e_herzog_g@ukw.de; 3 Department of Internal Medicine, Division of Hepatology, University Hospital Wuerzburg, University of Wuerzburg, 97080 Wuerzburg, Germany; geier_a2@ukw.de; 4 Comprehensive Heart Failure Center, University Hospital Wuerzburg, University of Wuerzburg, 97080 Wuerzburg, Germany; sequeira_v@ukw.de (V.S.); nickel_a@ukw.de (A.N.); kohlhaas_m@ukw.de (M.K.)

**Keywords:** MASH, MASLD, semaglutide, neuropeptide Y, PYY, obesity, diabetes, DIO rats

## Abstract

(1) Background: Modulators of the Neuropeptide Y (NPY) system are involved in energy metabolism, but the effect of NPY receptor antagonists on metabolic-dysfunction-associated steatotic liver disease (MASLD), a common obesity-related comorbidity, are largely unknown. In this study, we report on the effects of antagonists of the NPY-2 receptor (Y2R) in comparison with empagliflozin and semaglutide, substances that are known to be beneficial in MASLD. (2) Methods: Diet-induced obese (DIO) male Wistar rats were randomized into the following treatment groups: empagliflozin, semaglutide ± PYY_3-36_, the Y2R antagonists JNJ 31020028 and a food-restricted group, as well as a control group. After a treatment period of 8 weeks, livers were weighed and histologically evaluated. QrtPCR was performed to investigate liver inflammation and de novo lipogenesis (in liver and adipose tissue). Serum samples were analysed for metabolic parameters. (3) Results: Semaglutide + PYY_3-36_ led to significant weight loss, reduced liver steatosis (*p* = 0.05), and decreased inflammation, insulin resistance, and leptin levels. JNJ-31020028 prevented steatosis (*p* = 0.03) without significant weight loss. Hepatic downregulation of de novo lipogenesis-regulating genes (SREBP1 and MLXIPL) was observed in JNJ-31020028-treated rats (*p* ≤ 0.0001). Food restriction also resulted in significantly reduced weight, steatosis, and hepatic de novo lipogenesis. (4) Conclusions: Body weight reduction (e.g., by food restriction or drugs like semaglutide ± PYY_3-36_) is effective in improving liver steatosis in DIO rats. Remarkably, the body-weight-neutral Y2R antagonists may be effective in preventing liver steatosis through a reduction in de novo lipogenesis, making this drug class a candidate for the treatment of (early) MASLD.

## 1. Introduction

The increasing worldwide prevalence of metabolic-dysfunction-associated steatotic liver disease (MASLD) is a major health issue due to the associated increase in morbidity and mortality, but limited treatment options [1]. A recent meta-analysis states a global prevalence of 38% with an increase of 50% since 1990 [2]. Besides its close association to extrahepatic diseases within the spectrum of metabolic syndrome, such as type 2 diabetes mellitus (T2DM), hypertension, and cardiovascular disease, MASLD patients regularly have an increased all-cause mortality [3]. A progression of the disease results in increased inflammation and fibrosis, consequently leading to metabolic-dysfunction-associated steatohepatitis (MASH), liver cirrhosis, and hepatocellular carcinoma [4].

The key component of MASLD is liver steatosis. An accumulation of lipids in the liver may be caused by four different mechanisms: an increased hepatic uptake of circulating fatty acids from the blood stream, an increased de novo lipogenesis (DNL) in the liver, a decreased hepatic beta-oxidation, or a decreased hepatic lipid export [5]. Using labelled triglycerides (TGs), Donnelly et al. were able to demonstrate that 59% of TGs in the liver of MASLD patients were derived from circulating non-esterified fatty acids (NEFA) in the blood stream, 26% were derived from hepatic DNL, and 15% from diet [6]. The accumulation of lipids within the liver results in an environment of lipotoxicity, including mitochondrial dysfunction, endoplasmic reticulum stress, and apoptosis [7]. A central mechanism connecting obesity, T2DM, and MASLD is insulin resistance (IR) [8]. IR leads to lipolysis in white adipose tissue with subsequent uptake of fatty acids in the liver [9] and an increased hepatic DNL [5,10]. The adiponectin–leptin ratio is an indicator for adipose tissue dysfunction, and it was shown to be higher in individuals with greater insulin sensitivity [11]. The IR-induced disruption of insulin signalling leads to a preserved stimulation of lipogenesis (e.g., through activation of SREBP1), while the suppression of gluconeogenesis is not maintained (e.g., through PI3K, FoxO1 and PKB) [12,13].

Despite the increasing knowledge about MASLD and the obvious clinical significance, no therapeutic agent has been approved so far [14]. Weight loss is known to be beneficial for patients with MASLD. Bariatric surgery showed positive effects that may progress beyond simple weight loss as increased levels of metabolically beneficial gut hormones were detected [15,16]. Anti-obesity drugs like the glucagon-like peptide-1 (GLP-1) agonists liraglutide and semaglutide improve hepatic steatosis, possibly though an mTOR- and AMPK-mediated pathway [17,18]. The sodium glucose transporter 2 (SGLT2) inhibitor empagliflozin also showed positive effects on non-invasive measures of MASLD and liver fibrosis in humans without T2DM, while its molecular mechanisms promoting liver health are still not elucidated [19]. An activation of autophagy as well as reduced endoplasmic reticulum stress and apoptosis have been proposed as possible modes of action in a mouse model [20].

Further effective treatment strategies are clearly needed, and gut-derived hormones (like glucagon, GIP, etc.) as well as several neuropeptides may carry a potential to influence liver steatosis due to their food intake and energy-regulating characteristics. Neuropeptide Y (NPY) is an orexigenic neuropeptide that exerts its appetite-regulating effects in the hypothalamus. Different receptors (NPY receptor 1, 2, 4, 5, and 6) are known and partly mediate opposite actions. The NPY receptor 1 (Y1R) is mostly stimulated by intact NPY [21] and central stimulation of Y1R increases food intake [22], while antagonism reduces food intake [23,24]. Y2R is stimulated not only by NPY, but also by other peptides (e.g., the gut hormone PYY_3-36_) [21], and central Y2R stimulation reduces food intake [25], while a hypothalamic-specific knockout of Y2R resulted in increased food intake but decreased body weight, with sex-specific differences [26]. A treatment with the Y2R antagonist JNJ-31020028 in male rats demonstrated an exclusive high-fat diet (HFD) preference, while the body weights were not increased compared to controls [27]. Data on effects of modulators of the NPY system on liver steatosis are rare, but it was demonstrated that NPY knockout results in alleviated liver steatosis in HFD-induced obese mice [28]. Another study reported that Y2R deletion in hypothalamic neurons leads to an increased hepatic fat content only in female mice [29]. The gut hormone PYY_3-36_, which mostly exerts its beneficial effects through the Y2R, has shown its weight-reducing effects in multiple rodent [30,31], non-human primate [32], as well as in human studies [33]. A combination treatment with liraglutide and PYY_3-36_ improved liver steatosis in diet-induced obese rats [34].

This study aims to further investigate the actions of empagliflozin, semaglutide, PYY_3-36_, and the Y2R antagonist JNJ-31020028 on liver steatosis in a well-established HFD-induced obesity rat model.

## 2. Materials and Methods

### 2.1. Animals, Drugs and Treatment

The study design (including the weight course and HFD preferences) has been recently published [27]. In summary, obesity was induced in adult male Wistar rats (Charles River Laboratories, *n* = 34, 6 weeks old) using a HFD (4.615 kcal/kg; 45 kJ% fat, 20 kJ% protein, 35 kJ% carbohydrates) for 8 weeks. After diet-induced obesity was achieved (mean body weight 542 ± 13 g), the animals were randomized into the following treatment groups: [1] control group receiving a HFD (*n* = 6), [2] empagliflozin via drinking water (10 mg/kg/day; *n* = 5), [3] semaglutide s.c. daily (120 μg/kg/day; *n* = 6), [4] semaglutide s.c. daily (120 μg/kg/day) in combination with unselective NPY2R agonists (PYY_3-36_ 0.015 mol/kg/day, or NNC0165-0020; two animals also received the highly selective Y2R agonist NNC0165-1273) via osmotic minipump (*n* = 6), and [5] JNJ-31020028 (2.5 mg/kg/day; *n* = 6) via osmotic minipump. It has to be noted that a part of [5] (*n* = 3) also received exendin 9–39 (30 µg/kg/day) via osmotic minipump. Additionally, one food-restricted group [6] received 5 g/d of the above-mentioned HFD and 5 g/d of the mentioned low-fat diet (*n* = 5). Osmotic minipumps were implanted in the interscapular region (for [4]) or intraperitoneally (for [5]) under isoflurane anaesthesia with buprenorphine and carprofen analgesia. For interscapular implantation, a 1 cm incision was made, a subcutaneous pocket was formed using a sterile forceps, and the pump was inserted into this. For intraperitonal implantation, a 1 cm incision was made in the lateral abdomen and the pump was inserted intraperitoneally. During the treatment period, animals were weighed daily, single-housed, and had free choice of high-fat diet (HFD) and low-fat diet (3.630 kcal/kg; 10 kJ% fat, 20 kJ% protein, 70 kJ% carbohydrates). Additional information on the manufacturers of the used compounds can be found in the Supplementary Materials.

### 2.2. Dissection and Sample Collection

Blood samples pre-treated with dipeptidyl peptidase-4 inhibitor (Merck, Darmstadt, Germany) were taken directly from the abdominal aorta at the end of the experimental period under deep anaesthesia with isoflurane and butorphanol (2 mg/kg body weight). Immediately following blood sampling, the liver was removed and weighed.

### 2.3. Liver Histology

After defrosting, all samples were fixated in 10% neutral formalin for 24 h, using pieces of 1.0 × 0.5 cm and further prepared for paraffin embedding. Using a microtome, 3 µm cross-sections were cut. After de-paraffinization and rehydration, all samples received a haematoxylin/eosin and Sirius Red staining (500 aqueous picric acid solution 1.2% with 100 mg Sirius Red). Samples were assessed by a pathologist (GH) who was blinded regarding the treatment groups. Assessment of histological features of micro- and macrovascular steatosis, inflammation, and fibrosis was performed using the MASLD scoring system for rodent models as described by Liang et al. [35].

### 2.4. Enzyme-Linked Immunosorbent Assay and Serum Measurements

Plasma levels of insulin, leptin, adiponectin, and fructosamine were measured using rat-specific assays (details are found in the Supplementary Materials). Aspartate transaminase (AST), alanine transaminase (ALT), triglycerides (TG), cholesterol (CH), and non-esterified fatty acids (NEFA) were measured in an external laboratory (Laboklin, Bad Kissingen, Germany) using a Roche cobas 8000 modular analyser. The measurements were performed with commercial kits due to the manufacturer’s protocols. The applied test principles use photometric determination of products of enzymatic reactions of respective kits. Measurement of blood glucose was conducted twice per sample using handheld glucose meters. The average of two measurements was used for HOMA-index calculation. The HOMA index was calculated with fasting values for the individual rats as follows:

HOMA = insulin [mU/L] ∗ glucose [mmol/L]/22.5

### 2.5. Gene Expression Analysis

QrtPCR was used to investigate liver inflammation and to assess DNL in the liver and the visceral adipose tissue. In total, 10 mg liver tissue (or 100 mg visceral adipose tissue, respectively) was homogenized using a QIAGEN Tissue Lyser II (QIAGEN, Venlo, The Netherlands) and purified using Proteinase K Solution (Promega, Fitchburg, WI, USA). For liver samples, RNA was extracted using the Maxwell^®^ RSC simplyRNA Tissue Kit (Promega, Fitchburg, WI, USA). RNA of visceral adipose tissue was extracted using the QIAGEN RNeasy Plus Universal Mini Kit (QIAGEN, Venlo, The Netherlands) according to the manufacturer’s instructions. RNA concentrations were measured and tested for purity using a NanoDrop 2000c spectrophotometer (Thermo Fisher Scientific, Santa Clara, CA, USA). An RNA-integrity score (RIS) was obtained using the capillary gel electrophoresis system QIAxcel Connect (QIAGEN, Venlo, the Netherlands). If the RIS was below 5, RNA extraction was repeated. Liver RNA was reversely transcripted into cDNA using the QIAGEN QuantiTect Reverse Transcription Kit (QIAGEN, Venlo, the Netherlands), while visceral tissue RNA was reversely transcripted using ThermoFisher High-Capacity cDNA Reverse Transcription Kit with RNase Inhibitor (Thermo Fisher Scientific, Santa Clara, CA, USA) on an Eppendorf Mastercycler Gradient Instrument (Eppendorf SE, Hamburg, Germany). QrtPCR was performed on duplicates using TaqMan hydrolysis probes (Thermo Fisher Scientific, Santa Clara, CA, USA) of several preselected gene expression assays (TNF, IL1B, FGF21, SLC2A4, SREBP1, MLXIPL) on a Bio-Rad CFX96™ (Bio-Rad, Hercules, CA, USA). Duplicates with ∆Cq > 0.8 were excluded. Gapdh (used only in liver), Ubc (used only in fat) and beta-2 microglobulin (used only in fat) were used as reference genes. Reaction efficiency was calculated using LinRegPCR software (V. 2020.0). Efficiency-correction and normalization to multiple reference genes was conducted using qBase+ version 3.2 (Biogazelle, Gent, Belgium) based on normalization methods published by Hellemans et al. [36]. Through this calculation, normalized relative quantities (NRQs) were obtained. As NRQs are usually log-normal distributed [37], these values were log-transformed before statistical analysis.

### 2.6. Statistical Analysis

Statistical analyses were conducted using GraphPad Prism version 9.5.1 for Windows (GraphPad Software, La Jolla, CA, USA). Statistical significance was tested using one-way ANOVA with Holm–Sidak multiple comparison test for multiple comparison correction where appropriate. Two-way ANOVA with the Holm–Sidak multiple comparisons test were used for statistical analysis of changes in body weight over time. *p*-values < 0.05 were assumed to be significant.

## 3. Results

### 3.1. Body Weight Change

**Figure 1:**
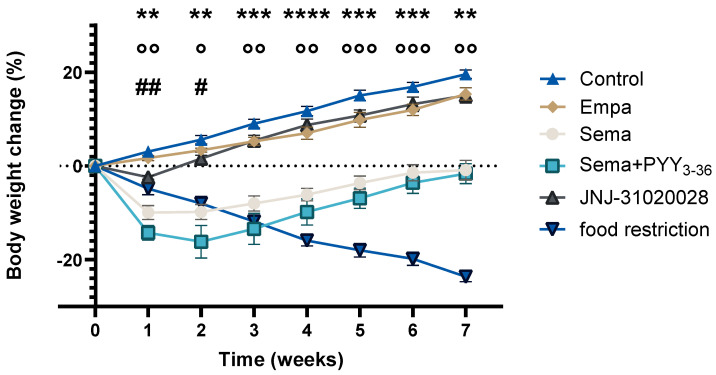
Weekly body weight change from baseline (in %) of the different treatment groups. The mean body weight of semaglutide-treated DIO rats differed significantly from the control group in weeks 1–7 (** *p* ≤ 0.01; *** *p* ≤ 0.001; **** *p* ≤ 0.0001). The mean body weight of semaglutide + PYY_3-36_-treated animals differed significantly from the control group in weeks 1–7 (° *p* ≤ 0.05; °° *p* ≤ 0.01; °°° *p* ≤ 0.001). The mean body weight of JNJ-31020028-treated animals differed significantly from the control group in weeks 1 and 2 (# *p* ≤ 0.05; ## *p* ≤ 0.01). The mean body weight of the food-restricted group differed significantly from the control group at week 1 (*p* ≤ 0.001), 2 (*p* ≤ 0.01) and weeks 3–7 (*p* ≤ 0.0001) (not indicated in the graph). Data are presented as mean ± standard error of the mean. Error bars that are not visible were too short to be mapped.

Figure 1 shows the weight course in different treatment groups. Control animals continued to gain weight, whereas a treatment with semaglutide led to a significant reduction in body weight at each time point. Weight loss was even more pronounced in the semaglutide and PYY_3-36_ combinatory-treated group. Treatment with JNJ-31020028 achieved a minor weight loss compared to the control group, which was significant only in the first two weeks. Empagliflozin-treated animals achieved only an insignificant weight loss compared to the control group.

### 3.2. Liver Weight and Histological Assessment: All Agonistic and Antagonistic Incretin-Based Treatments Improved Liver Steatosis Compared to a High-Fat Diet

**Figure 2:**
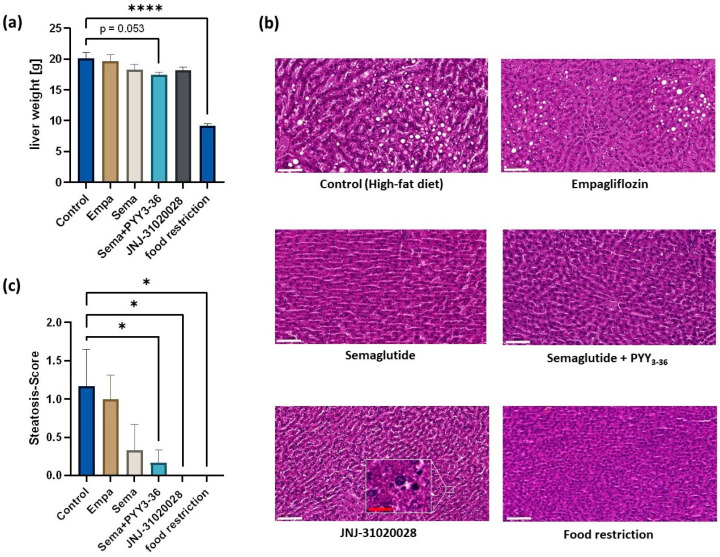
(**a**) Liver weights presented as mean ± standard error of the mean. Levels of significance: **** *p* ≤ 0.0001 (**b**) Examples of investigated liver samples in haematoxylin/eosin staining at 20× magnification, the in-frame picture was made at 100× magnification. White scales inserted on histologic slides represent 100 µm, red scale represents 20 µm. (**c**) Steatosis score in different treatment groups. Data are presented as mean ± standard error of the mean. Levels of significance: * *p* ≤ 0.05.

The liver weight was reduced significantly in the body-weight-matched group upon food restriction and reduced markedly in the semaglutide and PYY_3-36_ combinatory-treated group (*p* = 0.054, see Figure 2a). The blinded pathological assessment of liver steatosis revealed an improvement in all treatment groups compared to the control group as displayed in Figure 2b,c. Animals that were treated with semaglutide showed improved liver steatosis (mean MASLD score 0.33) compared to controls. A combinatory treatment with semaglutide and PYY_3-36_ induced a significantly reduced liver steatosis with a mean MASLD score of 0.17. Steatosis was completely resolved in the livers of all animals, that were treated with JNJ-31020028 (mean MASLD score 0.0). As an incidental finding, a granulocytic cytoplasm was detected in the livers of JNJ-31020028-treated animals. The calorie-restricted group also showed no steatosis (mean score 0.0).

### 3.3. Assessment of Inflammation Markers Using Histological Assessment and QrtPCR

**Figure 3:**
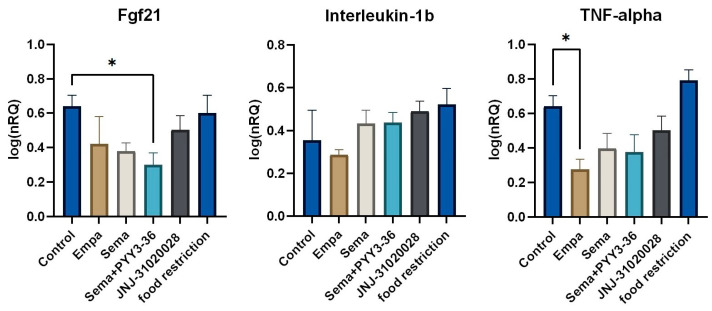
QrtPCR-based analysis of inflammation markers in liver tissue. Data are displayed as log-transformed normalized relative quantities (NRQs). Data are presented as mean ± standard error of the mean. * *p* ≤ 0.05.

In the blinded pathological assessment, no signs of advanced MASLD (e.g., inflammation or fibrosis) were detected in any rat. Molecular inflammation markers were analysed in the livers of the different treatment groups. Results are displayed in Figure 3. Fibroblast growth factor 21 (*Fgf21*) transcription was significantly reduced in semaglutide + PYY_3-36_-treated animals compared to animals in the control group (*p* = 0.024). Interleukin-1βb (*Il1b*) did not show any significant changes in transcription levels between different treatment groups. TNF-α (*Tnf*) transcription was significantly reduced in empagliflozin-treated animals compared to animals in the control group (*p* = 0.036).

### 3.4. Metabolic Measurements: Insulin Sensitivity Was Ameliorated and Leptin Was Decreased in Semaglutide and PYY_3-36_-Treated Animals, while Transaminases Did Not Show Any Differences

**Figure 4:**
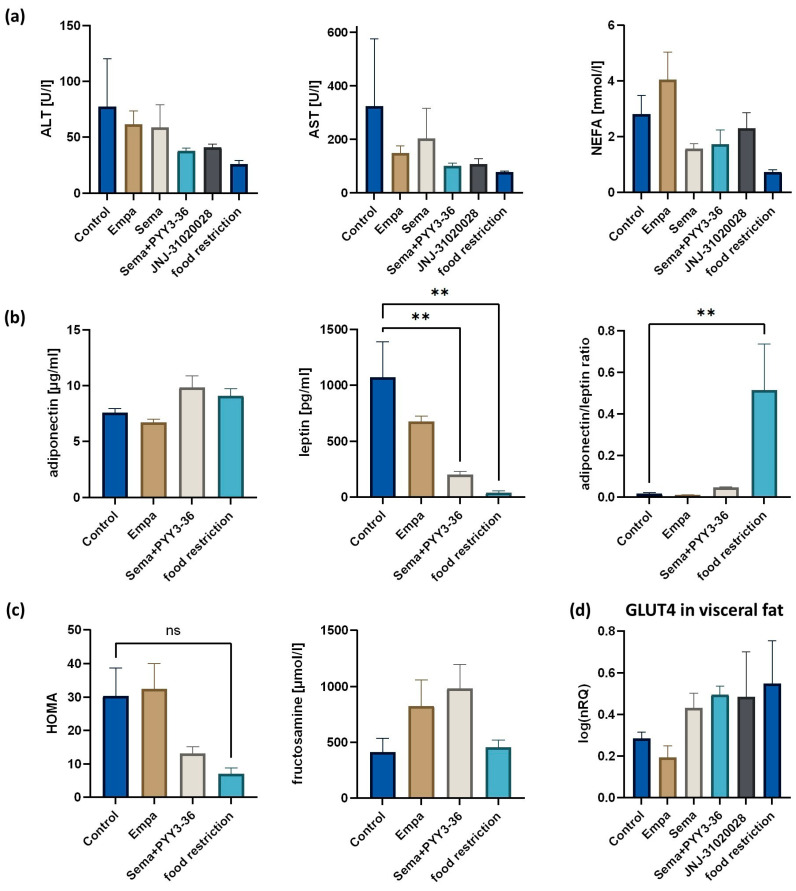
Metabolic measurements. (**a**) Serum levels of transaminases and non-esterified fatty acids (NEFA) in the serum of differently treated animals. Data are shown as mean ± standard error of the mean. ** *p* ≤ 0.01. (**b**) Adiponectin and leptin levels in the serum, as well as the calculated adiponectin–leptin ratio. Data are presented as mean ± standard error of the mean. ** *p* ≤ 0.01. (**c**) HOMA index and fructosamine measurements in the serum. ns = not significant. (**d**) QrtPCR-based analysis of GLUT4 expression in visceral adipose tissue. Data are displayed as log-transformed normalized relative quantities (NRQs). Data are presented as mean ± standard error of the mean. AST = alanine transaminase. ALT = aspartate transaminase. NEFA = non-esterified fatty acids. GLUT4 = glucose transporter type 4.

Metabolic measurements are displayed in Figure 4. Transaminases were measured to be lower in all treatment groups compared to the control group, but significance was not reached. Non-esterified fatty acids (NEFA) showed a trend towards an elevation in empagliflozin-treated animals and were decreased in all other treatment groups compared to the control group (Figure 4a). Triglycerides and cholesterol did not show any significant differences. No significant changes in adiponectin levels were detected. Leptin levels were significantly decreased in semaglutide + PYY_3-36_-treated animals (*p* = 0.004) and even stronger in the body-weight-matched group (*p* = 0.002), both compared to the control group (Figure 4b). The adiponectin–leptin ratio was only increased in the food-restricted group (*p* = 0.008). Insulin resistance was assessed via the HOMA index. The HOMA index was lower in semaglutide and PYY_3-36_ combinatory-treated animals (mean HOMA index 13.1 ± 4.2 vs. 30.3 ± 18.8 in the control group, *p* = 0.16), as well as in the body-weight-matched group (mean HOMA index 7.0 ± 3.7, *p* = 0.07) (Figure 4c). No significant changes in fructosamine levels were detected. To further explore insulin resistance, GLUT4 expression was measured in visceral fat. A trend towards an upregulation of the GLUT4 expression in the visceral fat of semaglutide ± PYY_3-36_-treated animals was observed. Additionally, the JNJ-31020028-treated, as well as the food-restricted, animals showed an upregulation of GLUT4 (Figure 4d).

### 3.5. Lipogenesis and Lipolysis in the Liver and the Visceral Adipose Tissue

**Figure 5:**
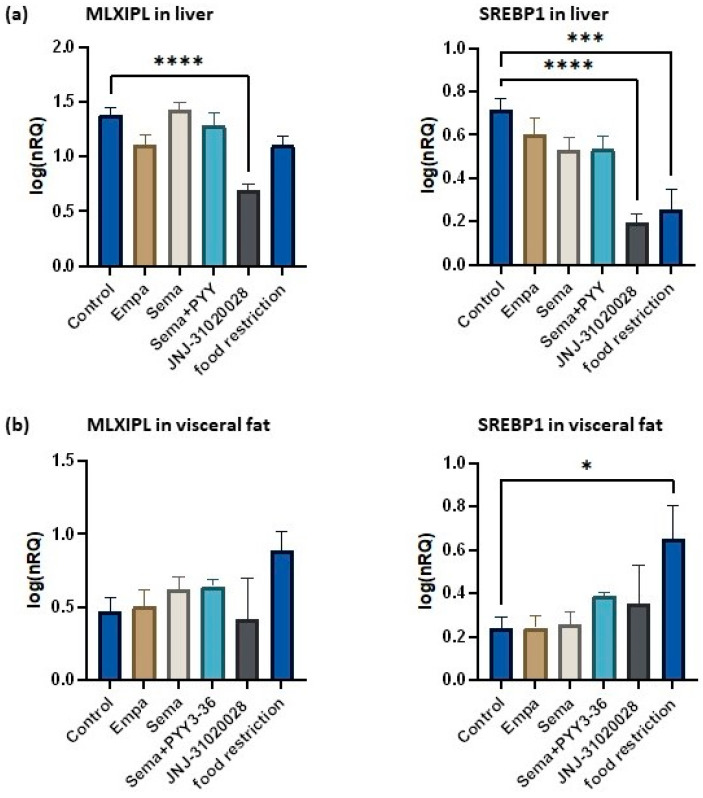
QrtPCR-based analysis of lipogenesis markers in liver (**a**) and visceral adipose tissue (**b**). Data are displayed as log-transformed normalized relative quantities (NRQs). Data are presented as mean ± standard error of the mean. * *p* ≤ 0.05, *** *p* ≤ 0.001, **** *p* ≤ 0.0001.

The liver samples and the visceral adipose tissue were analysed for genes that are involved in the regulation of lipogenesis. QrtPCR was used to investigate the expression of two known regulatory genes that induce de novo lipogenesis in the liver (SREBP1 and MLXIPL); results are displayed in Figure 5a. The analysis revealed a significant downregulation of SREBF1 and MLXIPL in the liver of rats treated with JNJ-31020028, compared to the control group. SREBP1 was significantly reduced in food-restricted animals as well (*p* < 0.001).

The visceral fat depots were analysed for the same lipogenesis-regulating genes (see Figure 5b). A significant upregulation of SREBP1 was found in the visceral fat of food-restricted animals compared to controls.

## 4. Discussion

In a randomised controlled study design, this study confirmed the positive effects of incretin agonists on liver health, while it provides evidence that Y2R antagonists are also beneficial in the treatment of liver steatosis. Animals treated with the Y2R antagonists JNJ-31020028 showed no steatosis at all, despite no changes in body weight and a remarkable preference for a HFD [27]. A downregulation of SREBP1 and MLXIPL was found, which points towards a reduced de novo lipogenesis in the liver of these animals, while lipogenesis in visceral fat was not affected. The exact mechanism of this action specific to the liver is not yet identified, but the histological observation that this treatment induced a hepatic cytoplasmic granulation clearly needs to be further investigated. In rodent studies using the Y2R antagonist BIIE0246, an increased hepatic glycogen accumulation was observed [38], which could possibly explain the granulation detected in this study.

The NPY system is strongly associated with the regulation of appetite and energy expenditure in the hypothalamus [39]. The Y2R is in the centre of NPY-associated anti-obesity research and, until recently, a central stimulation would have been assumed to reduce weight and be beneficial, while a blockage would have been assumed to increase body weight [25,26,40]. Despite its central, appetite-regulating actions, Y2R are also involved in oxidative fuel selection and lipid metabolism in peripheral tissues like fat, bones, and liver [41]. Genetic polymorphisms of the NPY gene (particullary the rs164147 polymorphism) have previously shown an association with insulin resistance and the development of steatohepatitis (the latter in obese subjects) [42,43]. As the JNJ-31020028-treated animals in the present study almost exclusively consumed a HFD [27] but did not develop liver steatosis, Y2R antagonists may be beneficial for liver health in an energy-rich setting as described before [38]. While this study reports on a downregulation of denovo lipogenesis-regulating genes after Y2R antagonisation, Chen et al. demonstrated that SREBP2 and 3-hydroxy-3-methylglutaryl-CoA reductase (HMGCR) are upregulated following direct administration of NPY into the portal vein [44]. These data support the conclusion that NPY has a negative impact on liver health by enhancing cholesterol biosynthesis. An indirect mechanism of action could possibly be found in the adipocyte–macrophage crosstalk, which is stimulated by NPY [28]. In the progression to hepatic fibrosis, NPY might also be a key player, as neprilysin-mediated cleavage of NPY resulted in fragments, that were held responsible for the activation of genes, which direct hepatic stem cells to develop towards fibroblast-like cells [45]. Interestingly, the administration of sacubitril, a neprilysin-inhibitor, in combination with valsartan, an angiotensin I receptor antagonist, led to a significant reduction in hepatic fibrosis and portal hypertension in mice [45]. The metabolic impact of the NPY system is still not fully understood, and the presented results clearly support further investigations in this interesting field.

Possible side effects of Y2R antagonists have not yet been investigated in humans, but animal studies revealed a number of possible modes of action that should be addressed in further studies. It was shown that Y2R knockout mice displayed a reduced anxiety like behaviour [46]. Alcohol-dependent rats showed a suppression of the motivation to self-administer ethanol upon treatment with the Y2R antagonist BIIE0246, which further strengthens the meaning of Y2R for CNS pathways [47]. While Y2R receptor agonists are most-likely anticonvulsant, it remains unclear whether Y2R antagonists may have pro-convulsive side effects [48]. Further studies showed effects on pain processing, while it remains unclear if NPY is pro- or antinociceptive [49]. Additionally, effects of Y2R antagonists on bone metabolism, cancer development, intestinal disease, and circadian rhythm disorder have been supposed [50].

In contrast to NPY antagonists, GLP-1 receptor agonists (e.g., semaglutide) are studied widely and showed positive effects on liver steatosis [17,18,34]. The exact mechanisms of action by which GLP-1 agonists induce liver-protection is not entirely clear, but recent studies suggested an involvement of the autophagy-lysosomal pathway [51,52]. In vitro, the GLP-1 agonist exendin-4 was suspected to enhance autophagic flux and reduce endoplasmatic reticulum stress, thereby executing antisteatotic effects through an activation of both macro- and chaperone-mediated autophagy [51,53]. The present study showed again that a polypeptide-based approach is more effective in inducing positive metabolic changes like weight-loss, reduced high-fat diet preference [27], and liver steatosis compared to a single peptide. In this setting, body weight loss may be regarded as a key component, since a food-restricted group that did not receive any pharmacologically active agent showed similar results regarding liver steatosis. The observed downregulation of *FGF-21* in livers of animals treated with semaglutide and PYY_3-36_ may provide a link between liver steatosis and inflammation [54], while advanced fibrotic stages of MASLD were not observed in the present study. Polypeptide approaches hold promise to soon improve MASLD treatment in clinical practice, since the first FDA-approved incretine-polypeptide tirzepatide (approved for the treatment of T2DM) has recently shown positive effects on the liver in a subpopulation of patients with T2DM [55]. Further developments of coagonists (e.g., GLP-1 and glucagon) may have additional beneficial effects on liver steatosis [56].

This study also showed an upregulation of SREBP1 in the visceral adipose tissue upon caloric restriction. It was described before that a reduction in leptin signalling may induce the expression of genes involved in lipogenesis (e.g., SREBP-1c) in adipocytes [57,58]. The capacity to expand adipose tissue on the other hand of course relies on the available substrates.

The beneficial effects of SGLT2 inhibition through empagliflozin on liver steatosis were not reproduced in this study. Liu et al. showed in mice that a treatment with the SGLT2 inhibitor dapagliflozin led to a reduced steatosis score and reduced expression of SREBP1c accordingly, possibly mediated by the activation of adenosine monophosphate-activated protein kinase (AMPK) phosphorylation and inhibition of the mammalian target of rapamycin (mTOR) phosphorylation pathways [59]. It was shown in ApoE-knockout mice that empagliflozin was able to attenuate MASLD progression, promote autophagy, and reduce endoplasmatic reticulum stress (as possible modes of action) [20]. In patients with T2DM, the SGLT2 inhibitor empagliflozin led to an improvement in the steatosis degree after a 6-month follow-up [60]. Despite possible interspecies differences, the animals in the present study only showed minor signs of insulin resistance that are not comparable with a manifest T2DM, which could at least explain in part why no major effects on MASLD were observed in empagliflozin treated rats. Furthermore, beneficial effects on liver steatosis may have been negated by the increased levels of NEFA in the present study. Recently, it was shown in humans that empagliflozin increased the uptake of free fatty acids in visceral adipose tissue by 27% (*p* < 0.05), while GLUT4 protein (*p* = 0.03) and mRNA content (*p* = 0.01) was reduced in abdominal s.c. adipose tissue without affecting glucose uptake, which clearly shows that empagliflozin may have pleiotropic effects that surpass its actions in the kidney [61]. Whether SGLT2 inhibitors could have beneficial effects in MASLD subjects without T2DM remains unclear.

## 5. Limitations

This study has several limitations. First, due to a too-short treatment period, rats exposed to a HFD did not develop advanced fatty liver disease stages with severe inflammation or marked fibrotic changes. Therefore, the used animal model can only be seen as a model of early MASLD. However, this can also be seen as an advantage, and effects on these early stages can be studied. Second, insulin resistance (assessed by HOMA index and fructosamine) was not markedly present in the investigated rodent model and functional glucose tolerance measurements were not performed. Third, following the 3Rs-principle, the numbers of animals per study groups in this exploratory pilot study were kept small, balancing animal suffering and the significance of results. Three of the animals in the Y2R antagonist (JNJ-31020028) group received a GLP-1 antagonist (exendin9–39) as well. We observed a steatosis score of 0.0 in all of the animals with similar changes in the observed molecular markers, so we assumed that the effect of exendin9–39 was negligible. Furthermore, two of the animals in the semaglutide and NPY agonist-treated group received a highly selective Y2R agonist (NNC0165-1273) in addition to an unselective NPY receptor agonist (PYY_3-36_ or NNC0165-0020), which had an initially pronounced effect on weight loss [27] but not on liver steatosis (after eight weeks of treatment). Fourth, transaminases and lipids in the blood were not altered in the animal model, but these parameters cannot be regarded as screening parameters for MASLD, as 80% of human MASLD patients have normal ALT levels [62]. Fifth, sex-specific aspects were not part of the study, but it is planned to repeat the experiments in female rats.

## 6. Conclusions

While the obesity pandemic continues to progress with increasing numbers of patients worldwide, effective treatment options for obesity-associated comorbidities like MASLD are urgently needed. Until now, the treatment of MASLD usually focusses on associated comorbidities, since specific treatment options are not yet available. This study underlines the relevance of weight loss in promoting liver health and gives further evidence that GLP-1 agonists like semaglutide induce not only weight loss, but also an improvement in early stages of MASLD. Polypeptide approaches (e.g., semaglutide in combination with PYY_3-36_) may be even more beneficial. Although not promoting long-term weight loss, the Y2R antagonist JNJ-31020028 has a positive impact on liver steatosis and reduces hepatic de novo lipogenesis. Following the presented data, Y2R antagonists need to be further evaluated as possible drug candidates in the treatment of MASLD, regardless of underlying comorbidities. Moreover, Y2R antagonists might even be effective in non-obese patients, where a weight-reducing drug is not applicable. Further research should go into detail about the exact mechanisms of action of Y2R antagonists. A multiomics approach will shed light on enriched pathways in the liver and other organs of interest.

## Data Availability

Partial or complete datasets and data dictionary are available upon request to Dr Ulrich Dischinger at dischinger_u@ukw.de, to investigators who provide an institutional review board letter of approval. The data are not publicly available due to ongoing research.

## Supplementary Materials

The following supporting information can be downloaded at: https://www.mdpi.com/article/10.3390/nu16060904/s1, Table S1: Animals, Drugs and treatment; Table S2: Enzyme-Linked Immunosorbent Assay and serum measurements; Table S3: Gene expression analysis, Instruments; Table S4: Gene expression analysis, Chemicals; Table S5: Gene expression analysis, TaqMan Probes (Thermo Fisher Scientific, Santa Clara, CA, USA). The graphical abstract was created with biorender.com.

## Abbreviations

ALT	alanine transaminase
AST	aspartate transaminase
BWM	body-weight-matched
CH	cholesterol
DIO	diet-induced obese
DNL	de novo lipogenesis
GIP	gastric inhibitory polypeptide
GLP-1	glucagon-like Peptide-1
GLUT4	glucose transporter type 4
HFD	high-fat diet
HOMA	homeostasis model assessment
IR	insulin resistance
MASLD	metabolic-dysfunction-associated steatotic liver disease
MASH	metabolic-associated steatohepatitis
NEFA	non-esterified fatty acids
NPY	neuropeptide Y
NRQs	normalized relative quantities
PYY_3-36_	peptide YY 3-36
QrtPCR	quantitatively real-time polymerase chain reaction
RNA	ribonucleic acid
SGLT2	sodium glucose transporter 2
T2DM	type 2 diabetes mellitus
TG	triglyceride
Y1R	NPY-1 receptor
Y2R	NPY-2 receptor
