# Supplementary material for: Effects of NPY-2 Receptor Antagonists, Semaglutide, PYY3-36, and Empagliflozin on Early MASLD in Diet-Induced Obese Rats
Source: Nutrients. 2024 Mar 21;16(6):904. doi: 10.3390/nu16060904 (PMC10974407; doi:10.3390/nu16060904)
Supplement: Supplementary file 1 [file nutrients-16-00904-s001.zip › nutrients-2899121-supplementary.pdf]

Supplementary Table S1: Animals, Drugs and treatment

Material	manufacturer
empagliflozin	Boehringer Ingelheim, Germany
exendin 9-39	Abcam, Cambridge, UK
high fat diet	#D12451 HF, Ssniff, Germany
JNJ-31020028	Janssen, New Jersey, USA
low fat diet	#D12450J LF, Ssniff, Germany
NNC0165-0020	Novo Nordisk, Denmark
NNC0165-1273	Novo Nordisk, Denmark
Osmotic minipumps	pump model 2006, ALZET, Cupertino, USA
PYY3-36	Cayman Chemical, Ann Arbor, USA
semaglutide	Novo Nordisk, Denmark

Supplementary Table S2: Enzyme-Linked Immunosorbent Assay and serum measurements

Target	manufacturer
adiponectin	#80570; Crystal Chem, Elk Grove Village, IL, USA
fructosamine	#RTEB1805; AssayGenie, Dublin, Ireland
insulin	#90010; Crystal Chem, Elk Grove Village, IL, USA
leptin	#EK-003-17; Phoenix Pharmaceuticals, Burlingame, CA, USA

Supplementary Table S3: Gene expression analysis, Instruments

Instrument	manufacturer
QIAGEN Tissue Lyser II	#85300; QIAGEN, Venlo, The Netherlands
Maxwell® RSC Instrument	#AS4500; Promega, Fitchburg, WI, USA
NanoDrop 2000c spectrophotometer	Thermo Fisher Scientific, Santa Clara, CA, USA
QIAxcel Connect	QIAGEN, Venlo, the Netherlands
Mastercycler Gradient Instrument	Eppendorf SE, Hamburg, Germany
CFX96™ Real-Time PCR Detection System	Bio-Rad, Hercules, CA, United States

Supplementary Table S4: Gene expression analysis, Chemicals

Chemical	manufacturer
Proteinase K Solution	#MC5005; Promega, Fitchburg, WI, USA
Maxwell® RSC simplyRNA Tissue Kit	#AS1340; Promega, Fitchburg, WI, USA
QIAGEN RNeasy Plus Universal Mini Kit	#73404, QIAGEN, Venlo, The Netherlands
High-Capacity cDNA Reverse Transcription Kit with RNase Inhibitor	#4374966; Thermo Fisher Scientific, Santa Clara, CA, USA
QuantiTect Reverse Transcription Kit	#205314; QIAGEN, Venlo, The Netherlands

Supplementary Table S5: Gene expression analysis, TaqMan Probes (Thermo Fisher Scientific, Santa Clara, CA, USA)

Gene	ID	GenBankSeq
TNF	Rn99999017_m1	NM_012675.3
IL1B	Rn00580432_m1	NM_031512.2
FGF21	Rn00590706_m1	NM_130752.1

SLC2A4	Rn00562597_m1	NM_012751.1
SREBP1	Rn01495769_m1	NM_001276707.1
MLXIPL	Rn00591943_m1	NM_133552.1
GAPDH	Rn01775763_g1	NM_017008.4
UBC	Rn01499642_m1	BC103477.1
beta-2 microglobulin	Rn03928990_g1	FQ219455.1
